# Cost-Effectiveness of Inpatient Continuous Glucose Monitoring

**DOI:** 10.7759/cureus.55999

**Published:** 2024-03-12

**Authors:** David Veríssimo, Beatriz R Pereira, Joana Vinhais, Catarina Ivo, Ana C Martins, João N Silva, Dolores Passos, Luís Lopes, João Jácome de Castro, Mafalda Marcelino

**Affiliations:** 1 Department of Endocrinology, Hospital das Forças Armadas, Lisbon, PRT

**Keywords:** portugal, type 2 diabetes, hospitalized patients, continuous glucose monitoring, cost-effectiveness

## Abstract

Introduction

Our department conducted a retrospective cohort study to compare the efficacy of continuous glucose monitoring devices versus capillary blood glucose in the glycemic control of inpatient type 2 diabetes on intensive insulin therapy in a Portuguese hospital. The use of continuous glucose monitoring devices was associated with improved glycemic control, including an increased number of glucose readings within target range and reduced hyperglycemic events, being safe concerning hypoglycemias. This is the cost-effectiveness analysis associated with these results.

Aim

The primary objective was to compare the cost-effectiveness of achieving glycemic control, defined as the number of patients within glycemic goals, between groups. Secondary endpoints included cost-effectiveness analyses of each time in range goal, and each percentual increment in time in range.

Methods

We defined each glycemic goal as: “readings within range (70-180 mg/dL) >70%”, “readings below range (below 70 mg/dL) <4%”, “severe hypoglycemia (below 54 mg/dL) <1%”, “readings above range (above 180 mg/dL) <25%”, “very high glycemic readings (above 250 mg/dL) <5%”.

Results

Continuous glucose monitoring showed lower median cost per effect for the primary outcome (€11.1 vs. €34.9/patient), with lower cost for readings in range (€7.8 vs. €11.6/patient) and for both readings above range goals ("above 180mg/dL": €7.4 vs. €9.9/patient, and "above 250mg/dL": €6.9 vs. €17.4/patient).

Conclusions

There are no published data regarding the cost-effectiveness of continuous glucose monitoring devices in inpatient settings. Our results show that continuous glucose monitoring devices were associated with an improved glycemic control, at a lower cost, and endorse the feasibility of incorporating these devices into hospital settings, presenting a favorable cost-effective option compared to capillary blood glucose.

## Introduction

During the coronavirus disease 2019 (COVID-19) pandemic, to minimize the exposure of healthcare personnel, our hospital used intermittently scanned continuous glucose monitoring (CGM) technology to monitor glycemic control in patients with diabetes. This glucose monitoring method was similarly adopted by several other medical departments, having been allowed by the Food and Drug Administration for this purpose [1].

Most studies using CGM in an inpatient setting focused on evaluating its accuracy and feasibility, with promising results [2,3]. However, the results were unclear on whether CGM was superior to capillary blood glucose (CBG), the gold standard in glucose monitoring [4], in improving glycemic control.

At the end of the COVID-19 pandemic, and to address the gap in the literature, our department conducted a cohort study aiming to compare the efficacy of CGM devices (FreeStyle Libre 2®; Abbott Laboratories, Chicago, IL, USA) versus CBG in glycemic control of inpatient type 2 diabetes on intensive insulin therapy [5]. The resulting data suggested that CGM devices may be more effective for inpatient diabetes management than CBG monitoring. The use of CGM devices was associated with improved glycemic control, including an increased number of glucose readings within target range, and reduced hyperglycemic events, especially for values above 250 mg/dL [5].

Although these findings supported the use of CGM devices in the inpatient setting as a potential alternative to CBG monitoring, the cost-effectiveness of CGM devices, an essential consideration for healthcare providers, was not evaluated. Although some studies addressed the cost-effectiveness of CGM compared to CBG in an outpatient setting [6-9], there are no published data on the cost-effectiveness of CGM in hospitalized patients.

Therefore, we aimed to evaluate the cost-effectiveness of implementing CGM devices in an inpatient setting.

## Materials and methods

Research design

Retrospective cohort study aiming to evaluate the cost-effectiveness of FreeStyle Libre 2, a CGM device, compared to CBG on glycemic control in a population of adults with type 2 diabetes exclusively on intensive insulin therapy during hospitalization in a Portuguese military hospital. Data was obtained from a previous study conducted by our department [5].

Patient selection

This study included the entire population from the previous efficacy study [5]. We included all the patients with type 2 diabetes patients, 18 years or older, capable of oral feeding, hospitalized for at least seven days in general medicine or surgery wards between January and December 2021, and on intensive insulin therapy, splited into two groups, depending on the type of monitoring that was used, until a total of 30 patients was included in each group (FreeStyle Libre 2 device - CGM group, CBG - CBG group).

Study objectives

The primary objective was to compare the cost-effectiveness of achieving glycemic control, defined as the number of patients within glycemic goals, between groups. Since the control group had no blind CGM we only considered the glucose readings registered by the nurses for each group and excluded the remaining glucose readings obtained by CGM devices. We defined each glycemic goal as: "readings within range (70-180 mg/dL) >70%", "readings below range (below 70 mg/dL) <4%", "severe hypoglycemia (below 54 mg/dL) <1%", "readings above range (above 180 mg/dL) <25%", "very high glycemic readings (above 250 mg/dL) <5%". Only patients meeting all glycemic goals were considered to have glycemic control.

Secondary endpoints included cost-effectiveness analyses of achieving each glycemic goal, the cost-effectiveness for each percentual increment in readings within range, for increasing the number of daily readings, and for reducing inpatient glucose management costs.

Cost-effectiveness analysis

Cost-effectiveness was evaluated by comparing both groups’ average cost-effectiveness ratio (ACER). ACER was defined as: (mean cost)/(number of patients achieving a glycemic goal).

CGM and CBG costs included the costs supported by the patients related to acquiring each FreeStyle Libre 2 sensor (€53.0/sensor) and each CBG kit (€0.64/kit).

The cost associated with glucose management was calculated as the sum of the costs related to glucose monitoring, insulin administration and hypoglycemia management. Glucose monitoring costs included the cost associated with the time spent by the healthcare staff (nurses) in introducing each FreeStyle Libre 2 sensor, in each CGM reading, and in performing a CBG. Insulin administration costs included the cost of the total insulin units given to each patient during hospitalization, the cost of each insulin administration kit used, and the cost associated with the time spent by the healthcare staff in administering insulin. The long-acting insulin used in our protocol was glargine U100 (€0.0279/unit) and the short-acting insulin was glulisine (€0.0153/unit). Each insulin administration kit cost €0.1319, and included a needle, a syringe, and a cotton dressing. The time spent by the healthcare staff (nurses) was monitored and converted into an amount of €0.21/min, according to the average nurse salary in our hospital. Hypoglycemia management cost was the sum of each glucose kit (€1.0388/kit) and the cost associated with the time spent by the healthcare staff in reverting hypoglycemia.

Statistical analysis

Quantitative data were presented as means and standard deviation or medians and quartiles, according to their adaptation to a normal distribution, which was evaluated by the Shapiro-Wilk test. Comparisons of independent samples were performed using the Student's t-test after evaluation of homoscedasticity (Levene's test) or using the Mann-Whitney U test. Pairwise comparisons were performed by applying the paired t-test or the Wilcoxon test. Qualitative data were presented as absolute and relative frequencies, and associations between independent subgroups were analyzed using Fisher's exact test. The analysis was performed using SPSS software version 23® (IBM Corp., Armonk, NY, USA), with a significance level set to 5%.

## Results

Baseline characteristics are described in Table 1 and were similar between groups.

**Table 1:** Baseline characteristics CGM: continuous glucose monitoring; CBG: capillary blood glucose; DCSI: diabetes complications severity index. Plus-minus signs are means ± standard deviations; Square brackets are medians [quartiles].

Characteristic		CGM (n = 30)	CBG (n = 30)	p-value
Age, years		76.8 ± 6.9	74.4 ± 9.4	0.277
Male sex, n (%)		25 (83.3)	19 (63.3)	0.143
Diabetes duration, years		12 [2;22.8]	13.5 [6.3;23]	0.824
Diabetes ambulatory treatment, n (%)				
	Diet / Lifestyle measures	3 (10)	3 (10)	0.570
	Non-insulin medications	21 (70)	16 (53.3)	
	Basal insulin	3 (10)	6 (20)	
	Intensive insulin	3 (10)	5 (16.7)	
Diabetes complications, n (%)				
	Retinopathy	2 (6.7)	6 (20)	0.254
	Nephropathy	13 (43.3)	13(43.3)	1.000
	Neuropathy	1 (3.3)	2 (6.7)	1.000
	Cardiovascular disease	7 (23.3)	9 (30)	0.770
	Cerebrovascular disease	5 (16.7)	7 (23.3)	0.748
	Peripheral arterial disease	6 (20)	8 (26.7)	0.761
	Metabolic complications	0 (0)	2 (6.7)	0.492
DCSI		1 [0;4]	2 [0;5.3]	0.151
Admission Diagnosis, n (%)				
	Infectious	16 (53.3)	14 (48.3)	0.157
	COVID-19	5 (16.7)	11 (36.7)	
	Cardiovascular	9 (30)	6 (20.7)	
	Gastrointestinal	3 (10)	2 (6.9)	
	Oncologic	1 (3.3)	7 (24.1)	
	Metabolic	1 (3.3)	0 (0)	
Admission laboratory results				
	Hemoglobin, g/dL	12.6 [9.8;13.9]	11.7 [9.5;12.9]	0.174
	Glucose, mg/dL	142.5 [116.8;197]	185 [136.8;297.8]	0.054
	Glycated hemoglobin, %	7.1 [6.1;8.4]	7.2 [6.3;8.6]	0.700
	Creatinine, mg/dL	1.4 [0.9;2.6]	1.3 [0.9;2.3]	0.390
	Urea, mg/dL	66 [46;133.8]	69 [55;109]	0.853
	Total cholesterol, mg/dL	161.5 [122;200.8]	165.5 [134.8;185.5]	0.701
	Low-density lipoprotein, mg/dL	93.2 ± 48.9	98.6 ± 33.7	0.618
	High-density lipoprotein, mg/dL	46.7 ± 14.9	41.3 ± 13.7	0.146
	Triglycerides, mg/dL	112 [76;187.5]	126 [98;164.5]	0.383
	Albuminuria, mg/g	23.1 [8.1;102.7]	53.4 [9.5;126.9]	0.311
Corticotherapy, n (%)		13 (43.3)	12 (40)	1.000

There were no significant differences between groups in terms of the median ambulatory daily dose of basal insulin (p=0.002), or short-acting insulin (p<0.001).

CGM group had a significantly higher number of patients with glycemic control (10 vs. 2, p=0.021; Table 2), despite no observed differences between groups regarding most of glycemic goals, except for "readings above 250 <5%" (16 vs. 4, p=0.002). The percentage of readings within range and the number of daily readings were also higher in the CGM group (68.5% vs. 42.0%, p=0.003 and 6 vs. 4, p<0.001; Table 2).

**Table 2:** Efficacy comparison CGM: continuous glucose monitoring; CBG: capillary blood glucose. Plus-minus signs are means ± standard deviations; Square brackets are medians [quartiles].

		CGM	CBG	Difference	p-value
Glycemic control		10 (33.3)	2 (6.7)	+8	0.021
	Readings within range >70%	14 (46.7)	6 (20)	+8	0.054
	Hypoglycemia <4%	23 (76.7)	27 (90.0)	-4	0.299
	Severe hypoglycemia <1%	28 (93.3)	27 (90.0)	+1	1.000
	Readings above 180 <25%	15 (50.0)	7 (23.3)	+8	0.060
	Readings above 250 <5%	16 (53.3)	4 (13.3)	+12	0.002
	Readings within range, %	68.5 [44.5;81.8]	42.0 [27.8;63.5]	+26.5	0.003

The median cost of implementing CGM devices is significantly higher than CGB (€106 vs. €56.2, p=0.004), with no difference in the glucose management cost (€45.7 vs. €44.6, p=0.701), despite a lower total glucose monitoring cost (€139.4 vs. €206.0, p=0.012), as observed in Table 3. Primary and secondary outcomes are described in Table 4.

**Table 3:** Cost comparison CGM: continuous glucose monitoring; CBG: capillary blood glucose. Square brackets are medians [quartiles].

Cost analysis			CGM	CBG	Difference	p-value
Cost of implementation			106.0 [53;119.3]	56.2 [33.1;75.5]	+49.8	0.004
	Total CGM/CBG kits		63	3265	-	-
	Total cost of implementation, €		3339.0	2089.5	+1249.5	0.004
Glucose management cost			45.7 [30.6;68.4]	44.6 [35.3;53.9]	+1.1	0.701
	Total glucose monitoring cost, €		139.4	206.0	-66.7	0.012
		Total time spent, minutes	664	981	-317	0.012
		Median glucose daily readings, n	6 [5;11]	4 [4;5]	+2	<0.001
	Total insulin administration cost, €		1383.1	1408.6	-25.5	0.918
		Total glargine units, n	8754	9153	-399	0.600
		Total glulisine, n	11334	14994	-3660	0.040
	Total hypoglycemia cost, €		228.5	50.8	+177.7	0.107
		Total number of hypoglycemia, n	171	38	+133	0.107

**Table 4:** Cost-effectiveness outcomes ACER: average cost-effectiveness ratio; CGM: continuous glucose monitoring; CBG: capillary blood glucose.

ACER		CGM	CBG	Difference
Primary Outcome				
	Glycemic control	€11.1/patient	€34.9/patient	-€23.8/patient
Secondary outcomes				
	Readings in range >70%	€7.9/patient	€11.6/patient	-€3.65/patient
	Hypoglycemia <4%	€4.8/patient	€2.6/patient	+€2.2/patient
	Severe hypoglycemia <1%	€3.9/patient	€2.6/patient	+€1.3/patient
	Readings above 180 <25%	€7.4/patient	€9.9/patient	-€2.5/patient
	Readings above 250 <5%	€6.9/patient	€17.4/patient	-€10.5/patient

In the ACER comparison, CGM showed a lower median cost per effect for the primary outcome (€11.1 vs. €34.9/patient). As for secondary outcomes, intervention achieved lower ACER for "readings in range >70%" (€7.8 vs. €11.6/patient) and for both goals regarding readings above range ("above 180 mg/dL <25%": €7.4 vs. €9.9/patient, and "above 250 mg/dL <5%": €6.9 vs. €17.4/patient). CGM had a higher median cost regarding hypoglycemia goals.

## Discussion

The present study aimed to compare the cost-effectiveness of a CGM device (FreeStyle Libre 2) with a CBG device for managing glycemic control in inpatients with type 2 diabetes undergoing intensive insulin therapy. Our results showed that CGM was more cost-effective than CBG monitoring in achieving glycemic control. Specifically, when CGM use was tailored to specific glycemic outcomes, such as increasing readings within target range and reducing readings above it, these devices proved to be more cost-effective than CBG. However, regarding hypoglycemia goals, CGM was less cost-effective in reducing readings below 70 mg/dL and below 54 mg/dL, being less effective and more costly than CBG. In the previous efficacy study [5], CGM proved to be a more effective tool for inpatient diabetes management than CBG, being safe regarding hypoglycemia.

The present study’s findings confirm that CGM is more effective and cost-effective in achieving glycemic control in this population. There are no published data regarding the cost-effectiveness of CGM devices in inpatient settings. Other studies that focused on an outpatient comparison between CGM and CBG showed that CGM was associated with an incremental cost-effectiveness ratio (ICER) of £3684/Quality Adjusted Life Year (QALY) in a United Kingdom population [6], an ICER of R$39,692.67/QALY in Brazilian type 2 diabetes patients [7], and €180,553/QALY in a Spanish meta-analysis [8]. Unfortunately, the previous efficacy study [5] showed no difference regarding inpatient mortality, infection, or length of stay. Therefore, we could not use QALY to compare to similar studies. This led us to restrain to ACER analysis to evaluate the effect gained by implementing the intervention. Considering the increasing number of hospitalized diabetes patients and the high frequency of hyperglycemia observed in these, we can infer the importance of adding a cost-effective medical device in controlling hyperglycemia. In Portugal, 140,339 patients with diabetes were hospitalized in 2021, representing 20.3% of all admitted patients and costing 310 million euros [10].

Additionally, hyperglycemia frequently occurs in these patients, which is associated with a high risk of complications and mortality [11], including volume and electrolyte disturbances mediated by osmotic diuresis, caloric and protein losses in under-insulinized patients, and higher susceptibility to infection [12].

Thus, our findings regarding the superiority of CGM in both efficacy and cost-effectiveness are of great relevance, adding evidence to the literature and eventually paving the way for other studies, including randomized clinical trials.

Limitations to our study include those described in the previous efficacy study [5], such as being a retrospective, single-center cohort study, with a relatively small sample size.

While retrospective studies may not establish causality as effectively as prospective ones, they offer valuable insights into real-world scenarios. Notably, the sample size of 60 patients divided into two groups is relatively small, potentially limiting the generalizability of findings to a broader population of inpatients with type 2 diabetes on intensive insulin therapy.

In addition to direct costs related to glucose monitoring, insulin administration, and hypoglycemia management, indirect costs and long-term implications must be considered. While focusing primarily on direct costs, such as device expenses and healthcare personnel time, we acknowledge the importance of assessing potential reductions in complications, readmissions, and overall healthcare utilization to provide a comprehensive cost-effectiveness evaluation in future studies.

Furthermore, the study recognizes the exclusion of glucose readings obtained by CGM devices, which may introduce bias by limiting the comparison between CGM and CBG groups. This exclusion highlights the continuous nature of CGM data compared to intermittent CBG readings, emphasizing the need for further exploration into its impact on study outcomes.

Regarding glycemic control, the study defines thresholds based on specific percentages of readings within certain ranges. While acknowledging the potential variability in optimal glycemic control thresholds across patients, the study provides valuable insights into the effectiveness of CGM in achieving these goals.

Notably, the study reports a higher median cost associated with CGM devices compared to CBG. However, we should not overlook the potential long-term cost savings and benefits associated with CGM, such as reduced need for intensive monitoring, fewer hypoglycemic events, improved clinical outcomes and additional benefits.

Finally, we acknowledge the importance of future studies providing comparative outcomes beyond glycemic control, such as patient satisfaction, quality of life, and clinical endpoints like hospital length of stay or readmission rates, to offer a more comprehensive assessment of the interventions' value.

## Conclusions

Our study suggests that the use of CGM devices may be more cost-effective for monitoring inpatient glycemic control than CBG. CGM devices were associated with an improved glycemic control, mainly reducing hyperglycemia, at a lower cost.

These findings support the feasibility of incorporating these devices in hospital settings, presenting a favorable, cost-effective option compared to CBG monitoring.
